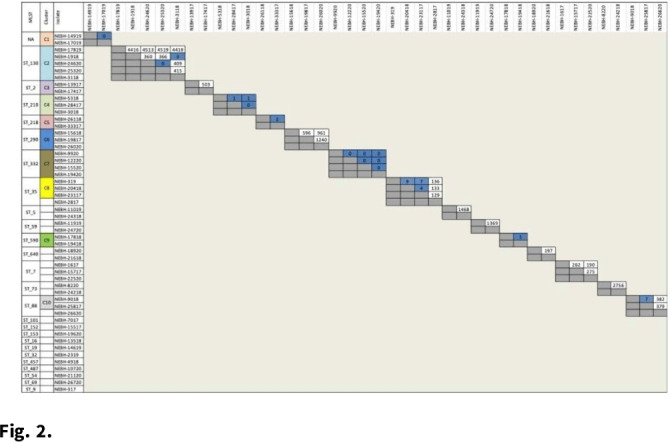# Whole-genome sequencing to assess clonality in a series of prosthetic joint *Staphylococcus epidermidis* isolates – WITHDRAWN

**DOI:** 10.1017/ash.2022.160

**Published:** 2022-05-16

**Authors:** Samantha Simon, Mohamad Sater, Ian Herriott, Miriam Huntley, Brian Hollenbeck

## Abstract

**Background:** Prosthetic joint infections (PJIs) are costly and cause increased morbidity and mortality for patients. *Staphylococcus epidermidis* is a common cause of both early postoperative and late-presenting PJIs. Although *S. epidermidis* is a normal part of the human skin microflora, its ability to form biofilm on implanted medical devices make it an important causative pathogen of PJIs. We investigated genetic, epidemiologic, and environmental factors contributing to *S. epidermidis* PJIs by performing whole-genome sequencing and clinical epidemiologic investigation of isolates collected from infected patients between 2017 and 2020. **Methods:** Patients with *S. epidermidis* isolated from a prosthetic joint that was placed at our orthopedic specialty hospital were identified using the microbiology laboratory records and electronic medical records. Whole-genome sequencing and single-nucleotide polymorphism (SNP)–based clonality analyses were performed using the epiXact service at Day Zero Diagnostics. These analyses included species identification, in silico MLST typing, phylogenomic analysis, as well as genotypic assessment of the prevalence of specific antibiotic resistance genes, virulence genes, and other relevant genes. For clonal isolates, additional reviews of surgical history and clinical data were performed. **Results:** In total, 62 *S. epidermidis* joint isolates were identified from 46 patients. Among these isolates, 52 were of sufficient purity to be used for genomic analysis (Fig. 1). A number of genes appeared in every isolate including *sepA*, *smr*, *cap*, *sesB*, *sesG*, and *embp*. Also, 6 *S. epidermidis* samples had a discrepancy between phenotypic resistance to oxacillin and the presence of the *mecA* resistance gene. We also identified 6 distinct clusters of isolates, all of which had SNP distances <10 base pairs (Fig. 2). Each cluster consisted of 2–4 patients. Cluster isolates accounted for 29.8% of all *S. epidermidis* prosthetic joint isolates. Most clonal isolates occurred in patients who were heavily exposed to different healthcare settings. Further epidemiologic investigation showed that some of these clonal isolates had ties to aspirations or procedures, whereas no clear connection could be determined for others. **Conclusions:** *S. epidermidis* isolated from clinical prosthetic joint samples contains a high degree of genetic resistance, including a mismatch between presence of *mecA* and phenotypic oxacillin resistance and genetic propensity for chlorhexidine resistance. Mupirocin resistance was not observed. Of all isolates, 29.8% belonged to multiple clusters, confirming hospital spread of this commensal organism in some cases.

**Funding:** None

**Disclosures:** None

**Figure:**
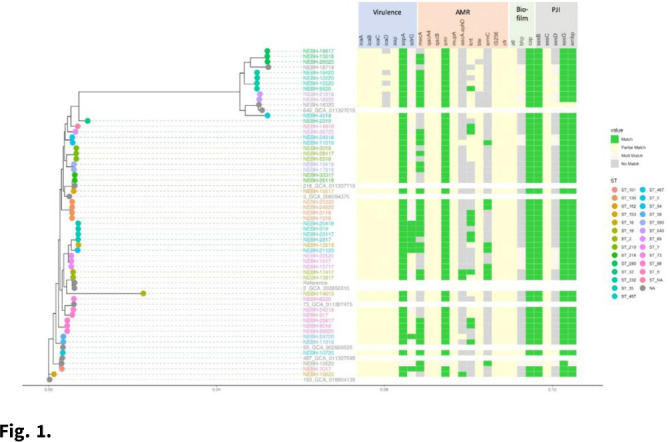

**Figure:**