# Whole-genome sequencing to assess clonality in a series of prosthetic joint Staphylococcus epidermidis isolates – WITHDRAWAL

**DOI:** 10.1017/ash.2022.309

**Published:** 2022-10-04

**Authors:** Samantha Simon, Mohamad Sater, Ian Herriott, Miriam Huntley, Brian Hollenbeck

This abstract was requested to be withdrawn by the authors prior to publication.

